# Secondary Syphilis With Isolated Ocular Syphilis and Superimposed Corneal Abrasion: A Case Report

**DOI:** 10.7759/cureus.92266

**Published:** 2025-09-14

**Authors:** Brandon C Stevens, Amna Zolj, John Abernathy

**Affiliations:** 1 Internal Medicine, HCA Florida Citrus Hospital/University of South Florida (USF) Morsani College of Medicine, Inverness, USA; 2 Internal Medicine, HCA Florida Brandon Hospital/University of South Florida (USF) Morsani College of Medicine, Brandon, USA

**Keywords:** corneal abrasion, neurosyphilis, ocular syphilis, secondary syphilis, syphilis, syphilitic uveitis

## Abstract

Ocular syphilis is a rare and easily preventable disease that can progress quickly to permanent blindness. Symptoms can mimic other ocular pathologies and further delay diagnosis. The incidence of syphilis continues to rise each year and represents significant morbidity in high-risk communities. In this case, the patient presented with unilateral blindness, uveitis, and a maculopapular rash on the lower extremities. Initially, she was found to have a corneal abrasion, leading to a missed diagnosis of ocular syphilis and delayed treatment. This case brings awareness to ocular syphilis as a key diagnosis to consider in a patient with unilateral ocular pathology that is refractory to treatment. Quick diagnosis is essential to prevent progression to permanent blindness.

## Introduction

Syphilis is a chronic multistage disease caused by a spirochete bacterium called *Treponema pallidum* (*T. pallidum*). It is transmitted through sexual contact or vertically from mother to child [1]. *T. pallidum* penetrates through microabrasions in the skin and intact mucous membranes [1, 2]. 

The incidence of primary and secondary syphilis has been increasing each year. From 2005 to 2013, reported cases in the United States nearly doubled, from 8,724 to 16,663 [3]. Most recent studies show an 11.2% increase from 2018 to 2019 [4]. Most diagnosed cases were among men and transgender women who have sex with men, with an increased prevalence in people with HIV [3,4].

Primary syphilis is a localized infection, manifesting as painless chancres at the site of inoculation, with regional lymphadenopathy. The incubation period is on average two to four weeks, with a range of nine to 90 days [1, 2]. The chancre erupts from a macule to a papule and finally an ulcer with a clean base and a flat, well-demarcated, and erythematous border, which can become indurated [1]. Chancres have also been reported as multiple and sometimes painful [2, 5]. Regional lymphadenopathy develops seven to 10 days after the chancre forms [2]. 

Secondary syphilis represents disseminated infection and can develop if initially untreated. The secondary stage develops three to 12 weeks after the appearance of the primary chancre [4, 6]. Presentation includes a diffuse and symmetric maculopapular rash on the trunk and extremities, classically also involving the palms and soles, mucocutaneous lesions, and lymphadenopathy [4]. The rash vastly ranges in appearance, observed to be macular, papular, pustular, or even nodular [4]. The rash is nonpruritic, nonpainful, and can have desquamation [6]. Other cutaneous findings include alopecia and condylomata lata-broad [6]. Syphilitic alopecia, described as having a "moth-eaten" appearance, may be the only presenting symptom, although it only occurs in 4% to 12.5% of cases [5].

Tertiary syphilis is a multiorgan disease that develops years to decades later. Cutaneous manifestations include noduloulcerative lesions, which tend to be more superficial, or gummatous lesions, which are destructive tumor-like rubbery nodules that tend to infiltrate deep into tissues, affecting any organ, and forming extensive fibrosis and scarring [7]. Cardiovascular syphilis occurs in 30% of cases and leads to aortitis, aortic aneurysm, and/or coronary ostiitis [7]. Late neurosyphilis presents with meningovascular lesions causing focal neurological deficits and parenchymatous lesions, leading to behavioral changes, memory deficits, general paresis, and tabes dorsalis [7, 8].

Ocular syphilis can occur at any stage and has the potential to affect every structure of the eye. It is a rare condition occurring in 1% of all syphilis cases [9]. Ocular syphilis is considered to be early neurosyphilis and can present with isolated ocular pathology without any other neurological deficits [10]. Panuveitis is the most common manifestation in HIV-positive patients, and posterior uveitis in HIV-negative patients [11]. Posterior segment and optic nerve complications include optic neuritis, intermediate and posterior uveitis, retinitis, chorioretinitis, neuroretinitis, retinal vasculitis, and acute syphilitic posterior placoid chorioretinitis [12]. Anterior segment complications include scleritis, episcleritis, interstitial keratitis (bilateral non-ulcerative corneal opacities), and anterior uveitis, which can present with anterior chamber cells, keratic precipitates, iris nodules, and posterior synechiae [9, 12]. Ocular syphilis may be accompanied by syphilitic meningitis, and CSF should be evaluated in patients who are having additional neurological symptoms [9, 10]. Diagnostic CSF analysis has no benefit in patients with isolated ocular syphilis without cranial nerve involvement [10]. Isolated ocular syphilis is a rare presentation of a reemerging disease. Early diagnosis has proven to be difficult due to its overlapping symptoms with other ocular pathologies. Here, we present a case of ocular syphilis without focal neurological deficits or cranial nerve involvement, suffering from a delayed diagnosis due to an initial finding of corneal abrasion. 

## Case presentation

This patient was a 48-year-old female with a past medical history of polysubstance abuse, fentanyl IV drug use, and trichomonas infection. She presented to the emergency department (ED) with left eye pain and erythema occurring for one month. She also noticed a diffuse maculopapular rash on the lower extremities bilaterally, which was not pruritic or painful. Approximately one week later, she began having significant left eye vision loss, described as only seeing shades. The patient denied eye trauma or contact use. 

The patient was previously seen at a different facility and was prescribed ophthalmic ofloxacin and steroid solutions for suspected corneal abrasion. Despite treatment over the course of seven days, there was no improvement in pain or erythema. She was also referred to an eye specialist, whom she admitted to not following up with. Upon returning to the ED with the same symptoms, the patient was evaluated further. 

**Figure 1:**
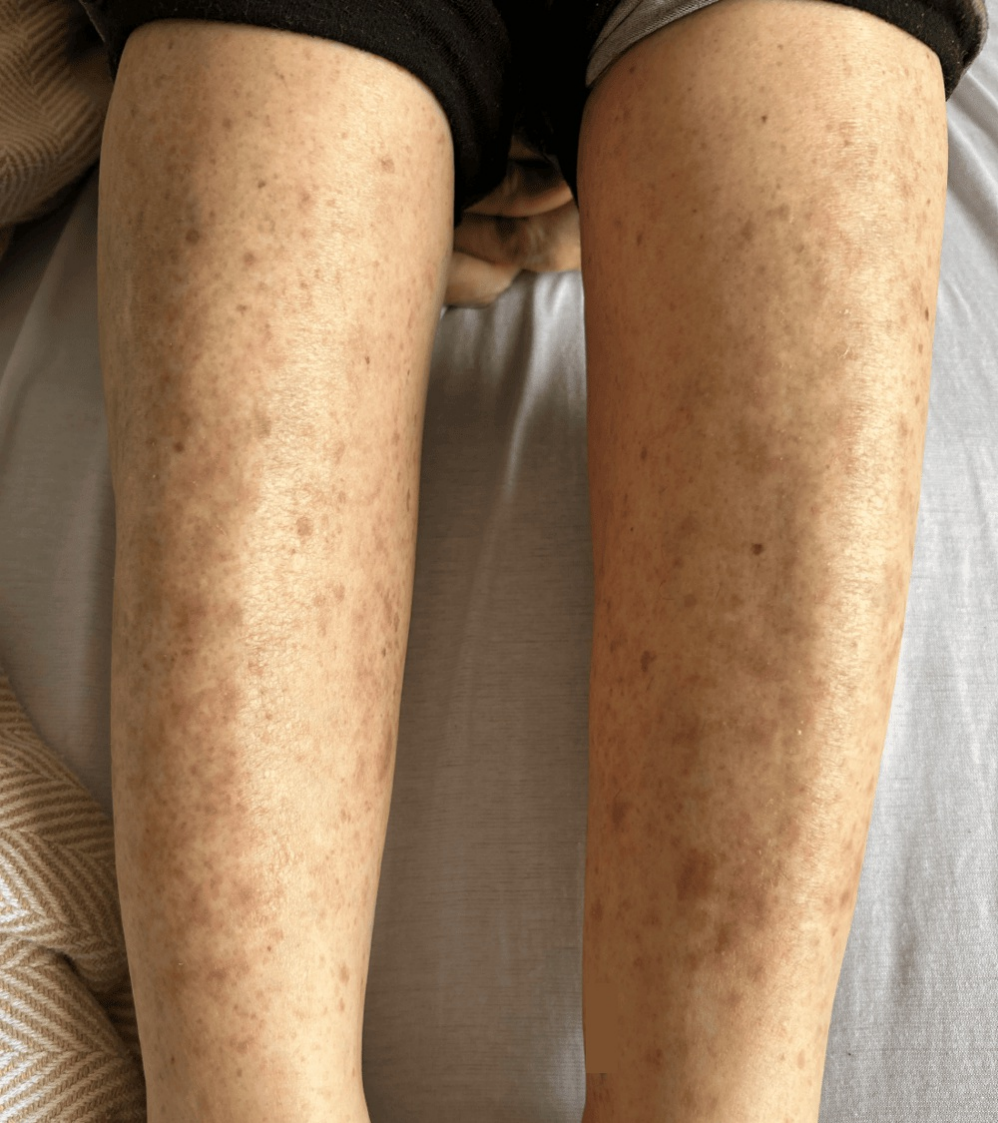
Maculopapular rash with mild desquamation on bilateral lower extremities

**Figure 2:**
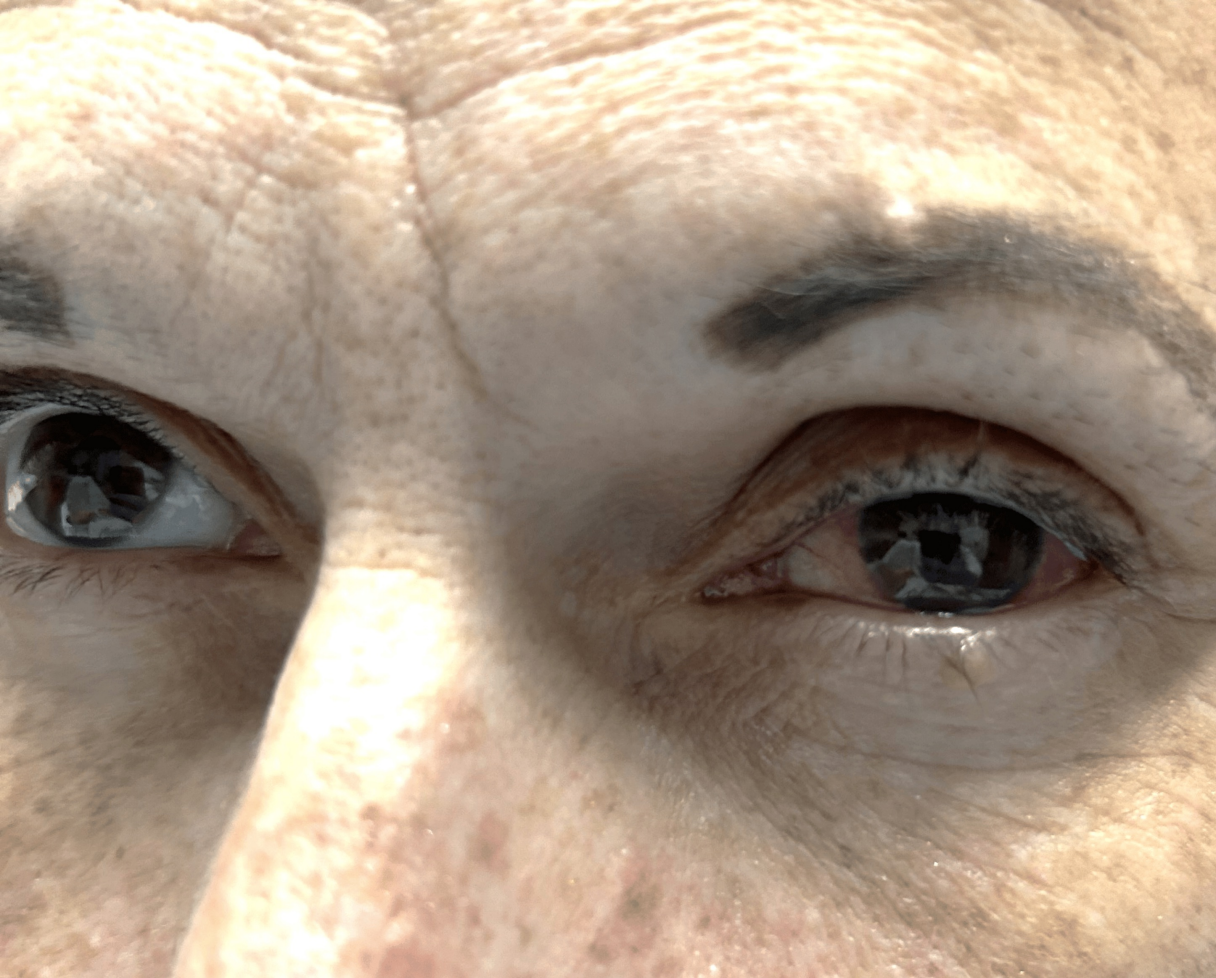
Circumcorneal injection of the left eye

Objectively, the patient was afebrile, normotensive, and tachycardic with a heart rate of 120 beats per minute (bpm). On physical exam, the lower extremities had a diffuse maculopapular rash bilaterally with mild desquamation (Figure 1). There was no periorbital swelling or tenderness with palpation. The patient did exhibit photophobia. On fundoscopic exam, there was retinal pigment epithelium (RPE) mottling. The slit lamp exam was significant for anterior chamber cells (1+). There was a left-sided circumcorneal injection (Figure 2), with tearing, and she had a slight left downward gaze. The pupil on the left had a sluggish pupillary reaction to light. Extraocular muscles were intact. Visual acuity of the left eye was presumed to be worse than 20/200, and the patient was unable to read any lines on the chart. The left eye was viewed with fluorescein and noted to have a corneal abrasion at the six o'clock position. Ocular pressure was read at 8 mmHg. An ultrasound of the left eye was obtained and showed no retinal detachment. Additionally, orbital CT with contrast revealed no inflammatory changes such as preseptal and postseptal cellulitis and no trauma or masses. 

It was then discussed with the patient to follow up with ophthalmology in the morning for further care and evaluation. The patient was given gentamicin eye drops. Rapid plasma reagin (RPR) and *T. pallidum* antigen (Ag) labs were sent out for testing, and she was discharged from the ED. Twenty days later, the patient was notified by the health department that she was positive for syphilis and to come to the hospital for treatment. 

The patient returned to the emergency department for treatment of syphilis. She denied any hearing loss, vertigo, weakness in the upper or lower extremities, ataxia, or paresthesia. Lab work was largely unremarkable, aside from syphilis testing, which showed RPR was positive at a titer of 1:128, and *T. pallidum *Ag was reactive. Her urine drug screen was positive for amphetamines and fentanyl. It was noted that she had an allergy to penicillin, so 2 g of IV ceftriaxone was administered. The patient was admitted for IV antibiotic treatment. The infectious diseases department was consulted. HIV, hepatitis panel, chlamydia, and gonorrhea labs were obtained as well. 

The next morning of admission, the patient was further questioned about her penicillin allergy, and it was reported as an erythematous rash without anaphylaxis. The patient was switched to the preferred treatment of aqueous penicillin G, 4 million units IV every four hours for 10 days. Over the course of the next 10 days, she reported improving rash, left eye pain, and erythema; however, her vision remained impaired. The patient was monitored for allergic reactions and never exhibited symptoms. Her hepatitis C antibody was positive; however, the ribonucleic acid polymerase chain reaction (RNA PCR) was not detected. Gonorrhea and chlamydia tests were negative. HIV testing was nonreactive.

## Discussion

The incidence of syphilis was previously thought to be decreasing due to the ease of treatment with the introduction of penicillin. However, since 2005, this disease has been reemerging [3,5]. Difficulty of diagnosis must be a contributing factor. Syphilis is a great imitator and has a wide range of manifestations. As discussed here, the patient may present mainly with ocular complaints that could be attributed to something benign, such as corneal abrasion. Although the patient had a corneal abrasion, she failed to improve after multiple visits to the emergency department and treatment with ophthalmic ofloxacin, gentamicin, and steroid solution. This patient did not have HIV and was not a man having sex with men. She did have an active sexual history and signs of secondary syphilis, such as the maculopapular rash, which helped guide us to the diagnosis. Ocular manifestations of uveitis and vision loss should prompt consideration of syphilis as a possible diagnosis. Diagnosis in a patient with an unknown history of syphilis should start with nontreponemal tests (NTT), either Venereal Disease Research Laboratory (VDRL) or RPR [7,10]. If NTT is nonreactive in a patient suspected to have early or latent syphilis, treponemal testing (TT) should be completed due to a potential false negative [13]. NTT is not exclusive to syphilis and can result in a false positive [14]. Additional confirmation is needed with TT, such as fluorescent treponemal antibody absorption (FTA-ABS) or T. pallidum particle agglutination assay (TPPA) [10,13]. A positive TT can be interpreted as an active infection or a previously treated infection and, therefore, must be supplemented with a detailed history and physical exam [14]. CSF analysis should be obtained if the patient has other neurological symptoms, cranial nerve involvement, or a history of neurosyphilis [10]. In this case, the patient did not exhibit any cranial nerve involvement according to physical exam and imaging findings. Some studies suggest that all patients with isolated ocular syphilis should have CSF analysis due to showing high CSF abnormality rates, although no benefit has been reported [15,16]. Ocular syphilis should be treated as neurosyphilis regardless of CSF analysis results [17]. Treatment for ocular syphilis is aqueous penicillin G, 4 million units IV every four hours for 10 to 14 days [17]. Another treatment option would be procaine penicillin G 2.4 million units intramuscularly (IM) once daily, plus probenecid 500 mg orally every six hours, both for 10 to 14 days [17]. If the patient has a penicillin allergy, desensitization or rechallenge should be performed [18]. An alternative would be ceftriaxone 1 to 2 g daily, either IV or IM, for 10 to 14 days; however, there is limited data to confirm its effectiveness for neurosyphilis [17]. The prognosis is extremely favorable depending on the degree of visual impairment when treatment is initiated [19]. With misdiagnosis and delayed treatment, the prognosis may be poor [19].

In this case, the patient had a corneal abrasion visualized with fluorescein stain. Her symptoms continued to worsen despite treatment. On reevaluation, there were findings of anterior chamber cells and circumcorneal injection suggestive of anterior uveitis. Additionally, there was retinal pigment epithelial irregularity and severely reduced visual acuity, consistent with posterior uveitis. Syphilitic uveitis is the probable mechanism for her acquired blindness. No keratic precipitates, vitreous cells, or interstitial keratitis were noted. No inflammation or masses were visualized on the CT orbit, and no retinal detachment on ultrasound. The intraocular pressure was within normal limits. The pupil was reactive and did not indicate significant ocular nerve damage. There was also the maculopapular rash on the lower extremities, characteristic of secondary syphilis. The patient had no focal neurological deficits or cranial nerve involvement to suggest neurosyphilis. Due to severe visual impairment at initial presentation, even with optimal treatment, visual acuity in the left eye did not improve. 

## Conclusions

This patient, who was initially treated for corneal abrasion, had a delayed diagnosis of ocular syphilis and was subsequently treated in the hospital with aqueous penicillin G, 4 million units IV every four hours for 10 days. After treatment, the patient’s lower extremity rash, left ocular pain, and left circumcorneal injection had completely resolved. However, her left eye blindness remained. This case study exemplifies the importance of early diagnosis and treatment, particularly in the setting of ocular syphilis.
